# Minimally invasive cardiac surgeries in 2023–annual report of the Japanese association for minimally invasive cardiac surgery

**DOI:** 10.1007/s11748-026-02383-8

**Published:** 2026-09-03

**Authors:** Tomoki Shimokawa, Hiraku Kumamaru, Noboru Motomura, Naoko Kinukawa, Hiroyuki Nishi, Hiroyuki Nakajima, Hiroyuki Kamiya, Kazuma Okamoto, Soh Hosoba, Yoshikatsu Saiki, Takashi Miura, Minoru Tabata, Akira Shiose, Taichi Sakaguchi

**Affiliations:** 1 Scientific Registry Committee of the Japanese Association for Minimally Invasive Cardiac Surgery, Tokyo, Japan; 2 Japan Cardiovascular Surgery Database Organization, Tokyo, Japan; 3 Department of Cardiovascular Surgery, Teikyo University, 2-11-1, Kaga, Itabashi-Ku, Tokyo, 173-8606 Japan; 4 Department of Health Data Science, Yokohama City University Graduate School of Data Science, Yokohama, Japan; 5 Department of Cardiovascular Surgery, Toho University Sakura Medical Center, Chiba, Japan; 6 Department of Cardiovascular Surgery, Tokai University Hachioji Hospital, Tokyo, Japan; 7 Department of Cardiovascular Surgery, Akita University, Akita, Japan; 8 Department of Cardiovascular Surgery, Asahikawa Medical University, Hokkaido, Japan; 9 Department of Cardiovascular Surgery, Hamamatsu University School of Medicine, Shizuoka, Japan; 10 Department of Cardiovascular Surgery, Nagoya Tokushukai General Hospital, Aichi, Japan; 11 Division of Cardiovascular Surgery, Tohoku University Graduate School of Medicine, Sendai, Japan; 12 Department of Cardiovascular Surgery, Nagasaki University, Nagasaki, Japan; 13 Department of Cardiovascular Surgery, Juntendo University, Tokyo, Japan; 14 Department of Cardiovascular Surgery, Kyushu University, Fukuoka, Japan; 15 Department of Cardiovascular Surgery, Hyogo Medical University, Hyogo, Japan

**Keywords:** Annual report, Minimally invasive cardiac surgery, Japanese association for minimally invasive cardiac surgery

## Abstract

**Objective:**

To establish updated nationwide perioperative benchmarks for minimally invasive cardiac surgery (MICS) in Japan by presenting the 2023 annual report and summarizing procedural trends from 2021 to 2023.

**Methods:**

Data on mitral valve (MV) surgery, aortic valve replacement (AVR), coronary artery bypass grafting (CABG), atrial septal defect (ASD) closure, and cardiac tumor resection performed via MICS in 2023 were extracted from the Japan Cardiovascular Surgery Database. Perioperative outcomes, including 30-day mortality, in-hospital mortality, conversion to median sternotomy, and major morbidity, were evaluated. Three-year trends in case volume, institutional distribution, procedural complexity, and early outcomes were descriptively reviewed.

**Results:**

The 2023 MICS cohort included the following cases: MV surgery (n = 3367), AVR (n = 1352), isolated CABG (n = 564), ASD closure (n = 232), and cardiac tumor resection (n = 126). Thirty-day and in-hospital mortality rates were 0.2% and 0.2%, respectively, in isolated MV repair, 1.3% and 1.9% in isolated MV replacement, 0.1% and 0.3% in isolated AVR, 0.9% and 1.2% in isolated CABG, 0% and 0% in ASD closure, and 0.8% and 1.6% in cardiac tumor resection. Among isolated procedures, the incidence of conversion to median sternotomy ranged from 0% to 2.6%. Over 2021–2023, case volume increased in all major categories, particularly in MV surgery and AVR, whereas MICS-CABG showed increasing complexity while maintaining acceptable early outcomes.

**Conclusions:**

Nationwide MICS activity in Japan has expanded progressively over the past 3 years in both volume and procedural scope, while generally favorable early outcomes have been maintained. These updated results provide an important benchmark for ongoing quality assessment in contemporary MICS practice.

## Introduction

Minithoracotomy-based open-heart surgery has become a widely adopted minimally invasive cardiac surgery (MICS) approach worldwide [1–3]. However, concerns regarding the learning curve and prolonged operative times remain [4]. In Japan, public health insurance reimbursement for sternal-sparing valve surgery began in 2018, resulting in an increasing number of institutions performing MICS procedures. To ensure procedural safety and establish real-world benchmarks at the national level, the Japanese Association for Minimally Invasive Cardiac Surgery (JMICS) has published annual outcome reports since 2021 [5, 6].

Cardiovascular surgery in Japan continues to evolve. According to the 2023 annual report of the Japanese Association for Thoracic Surgery (JATS), 63,427 cardiovascular surgeries were performed in 2023 (a 3.0% increase compared with 2022) [7], and the number of procedures for valvular heart disease increased by 3.2% to 17,805. Against this backdrop of increasing surgical volume, updated nationwide MICS data remain essential for ongoing quality assessment and benchmarking in contemporary clinical practice.

Building on previous findings, this study presents the 2023 annual report on mitral valve (MV) surgery, aortic valve replacement (AVR), coronary artery bypass grafting (CABG), atrial septal defect (ASD) closure, and cardiac tumor resection, using data from the Japan Cardiovascular Surgery Database (JCVSD), a nationwide registry of clinical information from nearly all hospitals performing cardiovascular surgery in Japan [8].

## Patients and methods

### Study population

Records of patients who underwent mitral valve (MV) surgery, aortic valve replacement (AVR), coronary artery bypass grafting (CABG), atrial septal defect (ASD) closure, or resection of a cardiac tumor via right or left minithoracotomy, thoracoscopic/port-assisted, or robotic-assisted approaches between January 1 and December 31, 2023, were obtained from the JCVSD within the National Clinical Database [8]. The dataset comprises approximately 300 variables (definitions available online at http://www.jacvsd.umin.jp), largely consistent with those of the Society of Thoracic Surgeons (STS) National Database (definitions available online at http://sts.org), and includes information on the surgical approach. Data registration and use for research purposes were approved by the Institutional Review Board of the National Clinical Database (16-041, August 29, 2016). The accuracy of the submitted data was verified through monthly random site visits by the site-visit working group [9].

A total of 63,427 cardiovascular surgeries performed in 2023 were registered in the JCVSD. Among 17,805 patients with valvular heart disease, 4719 underwent surgery via a right-lateral thoracotomy or a robotic approach. This MICS subset included 3367 MV cases (3019 repairs and 348 replacements) and 1352 aortic valve cases. Patients who underwent partial sternotomy (upper or lower hemi-sternotomy) or left thoracotomy were excluded, as were patients who underwent procedures without aortic cross-clamping, including those requiring circulatory arrest or ventricular fibrillation. The MV cohort included combined procedures such as tricuspid valve surgery, rhythm procedures such as the Maze procedure or pulmonary vein isolation, and aortic valve surgery.

To provide context, the national totals (all approaches) were 9131 for MV surgery and 9704 for aortic valve surgery in 2023. Accordingly, the estimated MICS penetration was 36.9% for MV surgery (3367/9131) and 13.9% for aortic valve surgery (1352/9704). These totals were derived from the JATS annual report and JCVSD aggregate data.

Among the registered cases, there were 14,454 CABG, 821 adult ASD, and 660 cardiac tumor cases in 2023. From these cohorts, the study included 564 CABG cases using a left-lateral approach, as well as 232 ASD and 126 cardiac tumor procedures using a right-lateral approach. Cases that did not meet the MICS approach criteria involving left or right minithoracotomy were excluded. The inclusion and exclusion criteria were identical to those used in the previous annual reports [5, 6], and the flowchart illustrating the study population selection was presented in the 2021 annual report [5].

### Study design

Preoperative variables and definitions of early postoperative outcomes were based on those previously defined by the STS. Thirty-day mortality was defined as death within 30 days of surgery, regardless of discharge status. In-hospital mortality was defined as death within any time interval following surgery in patients who were not discharged from the hospital. Postoperative length of stay was evaluated. Conversion to median sternotomy was defined as a case initially attempted via a right or left lateral approach but ultimately requiring a median sternotomy, as recorded in the JCVSD. The distribution of the number of MV surgeries per institution was also evaluated. Japan risk score for operative mortality (JapanSCORE) was used to evaluate the distribution of predicted mortality among patients [10].

### Statistical analysis

Data extraction and aggregation were performed by the data analysis team of the JCVSD (H.K., N.K.), and summary statistics were provided to the Scientific Registry Committee for this report. Continuous variables are presented as mean ± standard deviation or median (interquartile range [IQR]) as appropriate; length of stay in the intensive care unit and in hospital is summarized as the median because of its expected right-skewed distribution, whereas the remaining continuous variables are summarized as mean ± standard deviation, in accordance with the reporting format used in the previous annual reports [5, 6]. Categorical variables are presented as frequency (%). All analyses were descriptive, and no inferential statistical testing or between-group comparison was performed.

## Results

### Mitral valve surgery

The MICS-MV procedures included 3019 repairs (1879 isolated, 62.2%) and 348 replacements (154 isolated, 44.3%). The mean age was 59.2 ± 12.9 and 61.7 ± 12.8 years for isolated and overall MV repair, respectively, and 67.0 ± 14.8 and 69.5 ± 13.0 years for isolated and overall MV replacement (MVR), respectively. Urgent or emergent procedures included 114 (3.8%) MV repairs and 61 (17.5%) MVR cases. Infective endocarditis was present in 158 (5.2%) repair cases and 58 (16.7%) MVR cases. The mean JapanSCORE was 1.2 ± 2.2% for isolated MV repair and 7.1 ± 10.4% for isolated MVR. Concomitant procedures included tricuspid valve surgery (repair: 18.3%, MVR: 31.6%), arrhythmia surgery (repair: 17.9%, MVR: 27.0%), and aortic valve surgery (repair: 1.5%, MVR: 3.7%). The mean operative time was 271 ± 92 min for isolated MV repair, 281 ± 96 min for overall MV repair, 300 ± 266 min for isolated MVR, and 326 ± 135 min for overall MVR. Conversion to median sternotomy occurred in 26 (0.9%) overall MV repair and 12 (3.4%) overall MVR cases (Table 1).

**Table 1:** Preoperative and intraoperative characteristics of patients who underwent mitral valve surgery

	MV repair		MV replacement	
	Overall (n = 3019)	Isolated (n = 1879)	Overall (n = 348)	Isolated (n = 154)
Age (years)	61.7 ± 12.8	59.2 ± 12.9	69.5 ± 13.0	67.0 ± 14.8
Sex (male/female)	1918/1101	1227/652	154/194	67/87
Body surface area (m^2^)	1.66 ± 0.21	1.67 ± 0.21	1.55 ± 0.21	1.55 ± 0.21
Dialysis	25 (0.8%)	16 (0.9%)	12 (3.4%)	9 (5.8%)
Infective endocarditis (Active/treated)	158 (5.2%) (67/91)	120 (6.4%) (57/63)	58 (16.7%) (43/15)	47 (30.5%) (37/10)
Urgent/emergent	108/6	77/6	53/8	37/8
JapanSCORE (%)	–	1.2 ± 2.2	–	7.1±10.4
Operative time (min)	281 ± 96	271 ± 92	326 ± 135	300±266
CPB time (min)	185 ± 70	176 ± 66	215 ± 103	193±100
Cardiac arrest time (min)	124 ± 50	116 ± 46	141 ± 67	124±63
Transfusion	1345 (44.6%)	768 (40.9%)	271 (77.9%)	123 (79.9%)
IABP/PCPS	15/22	10/9	16/12	8/2
Tricuspid valve surgery	551 (18.3%)	–	110 (31.6%)	–
Arrhythmia surgery (Maze/PVI/ LAAC)	539 (17.9%) (340/47/420)	–	94 (27.0%) (39/8/70)	–
Aortic valve surgery	45 (1.5%)	–	13 (3.7%)	–
Conversion	26 (0.9%)	11 (0.6%)	12 (3.4%)	4 (2.6%)

JapanSCORE, Japan risk score for operative mortality; MV, mitral valve; CPB, cardiopulmonary bypass; IABP, intraaortic balloon pumping; PCPS, percutaneous cardiopulmonary system; PVI, pulmonary vein isolation; LAAC, left atrial appendage closure

The 30-day and in-hospital mortality rates were 0.2% and 0.2%, respectively, for isolated MV repair and 0.2% and 0.3% for overall MV repair. For MVR, these rates were 1.3% and 1.9%, respectively, for isolated cases and 2.6% and 3.4% for overall cases. Major postoperative morbidities in overall repair and overall MVR cases included re-exploration for bleeding (1.2% vs. 4.6%), stroke (0.7% vs. 4.0%), perioperative myocardial infarction (0.2% vs. 0%), aortic dissection (0.1% vs. 0.3%), and acute limb ischemia (0.1% vs. 0.3%). The median intensive care unit (ICU) and hospital stays were 2 and 10 days, respectively, for isolated MV repair, 2 and 11 days for overall MV repair, 3 and 16 days for isolated MVR, and 3 and 15.5 days for overall MVR (Table 2).

**Table 2:** Postoperative outcomes of patients who underwent mitral valve surgery

	MV repair		MV replacement	
	Overall (n = 3019)	Isolated (n = 1879)	Overall (n = 348)	Isolated (n=154)
30-day mortality	7 (0.2%)	4 (0.2%)	9 (2.6%)	2 (1.3%)
In-hospital mortality	10 (0.3%)	4 (0.2%)	12 (3.4%)	3 (1.9%)
Reoperation for bleeding	37 (1.2%)	24 (1.3%)	16 (4.6%)	7 (4.5%)
Stroke	22 (0.7%)	15 (0.8%)	14 (4.0%)	7 (4.5%)
Thoracotomy infection	4 (0.1%)	2 (0.1%)	1 (0.3%)	0
Groin area infection	1 (0.0%)	0	2 (0.6%)	0
Prolonged ventilation	46 (1.5%)	18 (1.0%)	28 (8.0%)	12 (7.8%)
Renal failure	35 (1.2%)	17 (0.9%)	18 (5.2%)	7 (4.5%)
New onset of AF	341 (11.3%)	204 (10.9%)	48 (13.8%)	15 (9.7%)
Heart block	27 (0.9%)	5 (0.3%)	5 (1.4%)	1 (0.6%)
Perioperative MI	5 (0.2%)	4 (0.2%)	0	0
Aortic dissection	2 (0.1%)	0	1 (0.3%)	1 (0.6%)
Acute limb ischemia	2 (0.1%)	2 (0.1%)	1 (0.3%)	1 (0.6%)
ICU stay (median, days)	2	2	3	3
Hospital stay (median, days)	11	10	15.5	16

MV, mitral valve; AF, atrial fibrillation; MI, myocardial infarction; ICU, intensive care unit; Underlined values denote medians

**Fig. 1:**
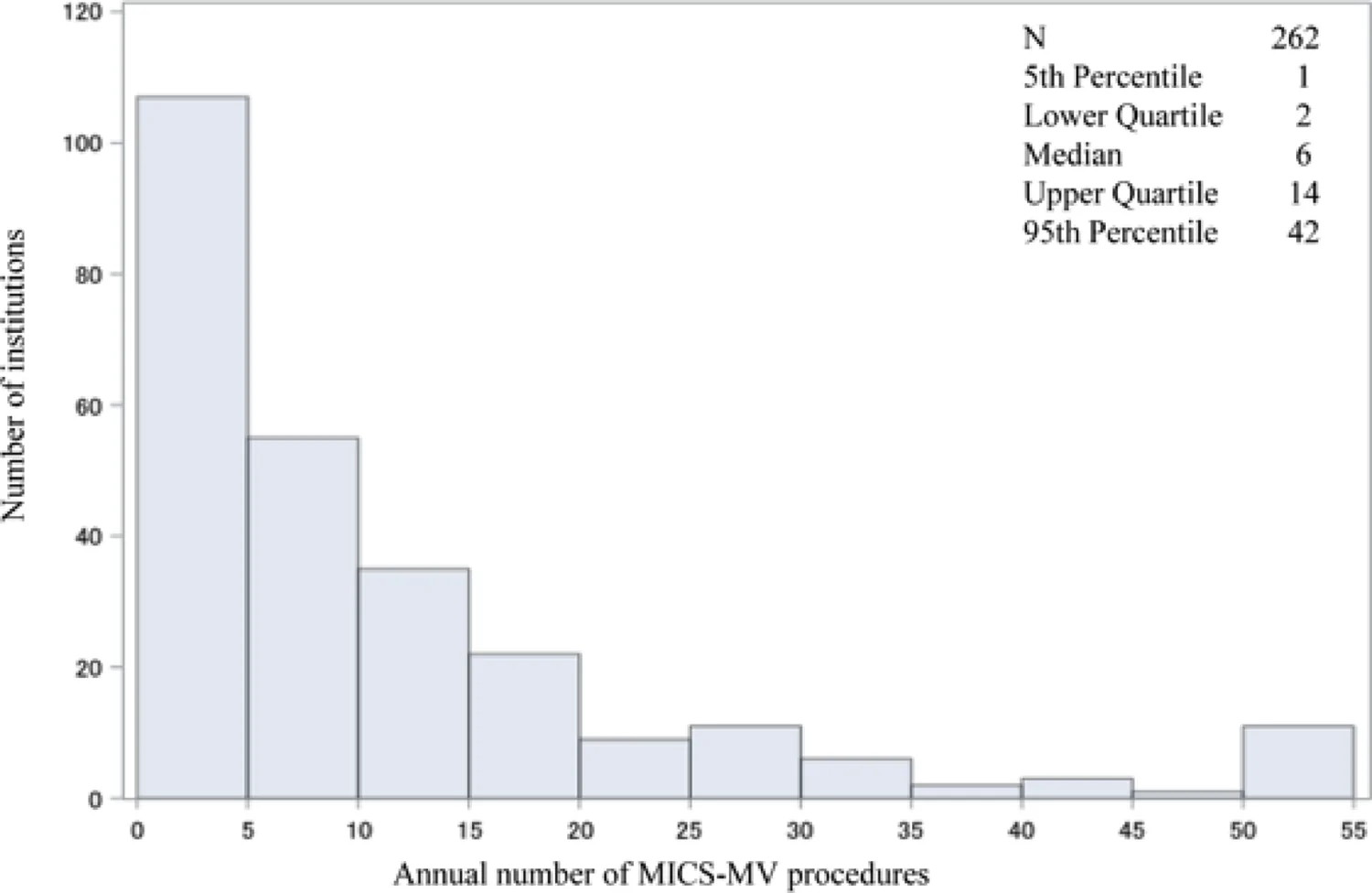
Institutional distribution of annual minimally invasive cardiac surgery mitral valve (MICS-MV) procedure volume. The histogram shows the distribution of institutions according to MICS-MV case volume in 2023. A total of 262 institutions performed MICS-MV procedures. The median annual case volume per institution was 6, with an interquartile range of 2 to 14

The distribution of MICS-MV procedures per hospital varied widely; among 262 institutions, more than 100 performed < 5 procedures per year (median: 6), although several institutions performed ≥ 50 procedures annually (Fig. 1).

### Aortic valve replacement

The MICS-AVR procedures included 1352 cases (1171 isolated cases, 86.6%). The mean age was 68.8 ± 11.8 years for isolated AVR and 69.2 ± 11.7 years for overall AVR cases. Urgent or emergent procedures were performed in 21 (1.8%) isolated and 25 (1.8%) overall AVR cases. Infective endocarditis was present in 16 (1.4%) isolated and 21 (1.6%) overall cases. The mean JapanSCORE was 2.7 ± 3.6% for isolated AVR. Concomitant procedures included tricuspid valve surgery in 21 (1.6%) and arrhythmia surgery in 62 (4.6%) cases. The mean operative time was 259 ± 88 min for isolated and 267 ± 94 min for overall cases. Conversion to median sternotomy occurred in 21 (1.8%) isolated and 31 (2.3%) overall cases (Table 3).

**Table 3:** Preoperative and intraoperative characteristics of patients who underwent aortic valve replacement

	All (n = 1352)	Isolated (n = 1171)
Age (years)	69.2 ± 11.7	68.8 ± 11.8
Sex (male/female)	838/514	726/445
Body surface area (m^2^)	1.62 ± 0.2	1.63±0.2
Dialysis	58 (4.3%)	52 (4.4%)
Infective endocarditis (Active/treated)	21 (1.6%) (8/13)	16 (1.4%) (7/9)
Urgent/emergent	23/2	20/1
JapanSCORE (%)	–	2.7±3.6
Operative time (min)	267 ± 94	259±88
CPB time (min)	162 ± 65	155±59
Cardiac arrest time (min)	109 ± 45	105±41
Mechanical/tissue valve/others	148/1193/11	130/1030/11
Transfusion	802 (59.3%)	676 (57.7%)
IABP/PCPS	9/6	3/4
Tricuspid valve surgery	21 (1.6%)	–
Arrhythmia surgery (Maze/PVI/LAAC)	62 (4.6%) (9/9/56)	–
Conversion	31 (2.3%)	21 (1.8%)

JapanSCORE, Japan risk score for operative mortality; CPB, cardiopulmonary bypass; IABP, intraaortic balloon pumping; PCPS, percutaneous cardiopulmonary system; PVI, pulmonary vein isolation; LAAC, left atrial appendage closure

The 30-day and in-hospital mortality rates were 0.1% and 0.3%, respectively, for isolated AVR and 0.2% and 0.5% for overall AVR. Major postoperative morbidities in isolated versus overall cases included re-exploration for bleeding (2.6% vs. 2.7%), stroke (0.8% vs. 1.0%), perioperative myocardial infarction (0.3% vs. 0.4%), and aortic dissection (0.2% vs. 0.4%). Acute limb ischemia occurred in 0.1% of cases in both groups. The median ICU stay was 2 days, and the median hospital stay was 11 days in both groups (Table 4).

**Table 4:** Postoperative outcomes of patients who underwent aortic valve replacement

	All (n=1352)	Isolated (n=1171)
30-day mortality	3 (0.2%)	1 (0.1%)
In-hospital mortality	7 (0.5%)	4 (0.3%)
Reoperation for bleeding	37 (2.7%)	30 (2.6%)
Stroke	13 (1.0%)	9 (0.8%)
Thoracotomy infection	0	0
Groin area infection	1 (0.1%)	0
Prolonged ventilation	19 (1.4%)	12 (1.0%)
Renal failure	27 (2.0%)	18 (1.5%)
New onset of AF	191 (14.1%)	158 (13.5%)
Heart block	16 (1.2%)	12 (1.0%)
Perioperative MI	5 (0.4%)	3 (0.3%)
Aortic dissection	5 (0.4%)	2 (0.2%)
Acute limb ischemia	1 (0.1%)	1 (0.1%)
ICU stay (median, days)	2	2
Hospital stay (median, days)	11	11

AF, atrial fibrillation; MI, myocardial infarction; ICU, intensive care unit; Underlined values denote medians

### Coronary artery bypass grafting

A total of 564 MICS-CABG procedures were performed. Diseased vessel distribution included left main trunk (LMT) lesions (n = 163), 3-vessel disease (n = 207), 2-vessel disease (n = 198), and single-vessel disease (n=140). Off-pump CABG was performed in 535 (94.9%) patients, and single-vessel bypass was performed in 356 (63.1%) patients. The left internal thoracic artery was used in 538 (95.4%) cases, followed by the right internal thoracic artery (n = 112), saphenous vein (n = 108), gastroepiploic artery (n = 42), and radial artery (n = 24). Conversion to median sternotomy occurred in 12 (2.1%) cases (Table 5).

**Table 5:** Preoperative and intraoperative characteristics of patients who underwent minimally invasive cardiac surgery coronary artery bypass grafting (MICS-CABG)

	Isolated (n = 564)
Age (years)	69.2 ± 11.7
Sex (male/female)	454/110
Body surface area (m^2^)	1.68 ± 0.19
Dialysis	70 (12.4%)
Urgent/emergent	77/5
JapanSCORE (%)	2.6 ± 3.7
Operative time (min)	247 ± 100
Transfusion	179 (31.7%)
IABP/PCPS	26/5
Number of diseased vessels	1VD:140, 2VD:198, 3VD:207, LMT:163, Unknown:19
Off pump vs on pump	Off pump: 535, On pump: 29
Number of distal anastomoses	1: 356, 2: 103, 3: 72, 4: 23, 5: 8, 6: 0, 7: 1, Unknown:1
Bypass graft	LITA: 538, SV: 108, RITA: 112, RA: 24, GEA: 42
Conversion	12 (2.1%)

JapanSCORE, Japan risk score for operative mortality; VD, vessel disease; LMT, left main trunk; LITA, left internal thoracic artery; RITA, right internal thoracic artery; SV, saphenous vein; GEA, gastroepiploic artery; RA, radial artery

The 30-day and in-hospital mortality rates were 0.9% and 1.2%, respectively. Postoperative morbidities included re-exploration for bleeding (0.9%), stroke (0.5%), and perioperative myocardial infarction (0.4%). The median ICU stay was 2 days and the median hospital stay was 11 days (Table 6).

**Table 6:** Postoperative outcomes of patients who underwent minimally invasive cardiac surgery coronary artery bypass grafting (MICS-CABG)

	Isolated (n = 564)
30-day mortality	5 (0.9%)
In-hospital mortality	7 (1.2%)
Reoperation for bleeding	5 (0.9%)
Stroke	3 (0.5%)
Prolonged ventilation	13 (2.3%)
Renal failure	12 (2.1%)
New onset of AF	46 (8.2%)
Perioperative MI	2 (0.4%)
ICU stay (median, days)	2
Hospital stay (median, days)	11

AF, atrial fibrillation; MI, myocardial infarction; ICU, intensive care unit; Underlined values denote medians

### Atrial septal defect closure and cardiac tumor resection

The mean age was 54.5 ± 17.4 years for ASD and 65.5 ± 13.9 years for cardiac tumor cases. Myxoma accounted for 91 (72.2%) of the tumors. In ASD cases, concomitant procedures included mitral valve surgery (n = 88, 37.9%), tricuspid valve surgery (n = 73, 31.5%), arrhythmia surgery (n = 37, 15.9%), and aortic valve surgery (n = 10, 4.3%). In tumor cases, concomitant procedures included mitral valve surgery in 12 (9.5%), arrhythmia surgery in 12 (9.5%), and tricuspid valve or aortic valve surgery in 2 cases (1.6%) each. The mean operative time was 252 ± 84 min for ASD and 253 ± 100 min for tumor cases (Table 7).

**Table 7:** Preoperative and intraoperative characteristics of patients who underwent atrial septal defect closure or cardiac tumor resection

	ASD (n = 232)	Cardiac tumor (n = 126)
Age (years)	54.5 ± 17.4	65.5 ± 13.9
Sex (male/female)	117/115	49/77
Body surface area (m^2^)	1.64 ± 0.20	1.60 ± 0.18
Dialysis	1 (0.4%)	3 (2.4%)
Infective endocarditis (Active/treated)	2 (0.9%) (1/1)	2 (1.6%) (1/1)
Urgent/emergent	1/0	11/4
JapanSCORE	–	–
Operative time (min)	252 ± 84	253 ± 100
CPB time (min)	155 ± 62	143 ± 63
Cardiac arrest time (min)	97 ± 47	76 ± 43
Transfusion	76 (32.8%)	66 (52.4%)
IABP/PCPS	0/1	0/0
Tricuspid valve surgery	73 (31.5%)	2 (1.6%)
Arrhythmia surgery (Maze/PVI/LAAC)	37 (15.9%) (16/8/34)	12 (9.5%) (4/2/9)
Aortic valve surgery	10 (4.3%)	2 (1.6%)
Mitral valve surgery	88 (37.9%)	12 (9.5%)
Myxoma	–	91 (72.2%)
Conversion	1 (0.4%)	0

JapanSCORE, Japan risk score for operative mortality; ASD, atrial septal defect; CPB, cardiopulmonary bypass; IABP, intraaortic balloon pumping; PCPS, percutaneous cardiopulmonary system; PVI, pulmonary vein isolation; LAAC, left atrial appendage closure

The 30-day and in-hospital mortality rates were 0% and 0%, respectively, for ASD and 0.8% and 1.6% for tumor cases. Postoperative morbidities in ASD versus tumor cases included new-onset atrial fibrillation (13.8% vs. 11.1%), re-exploration for bleeding (0.9% vs. 0.8%), and stroke (0% vs. 0.8%), with no perioperative myocardial infarctions in either group. The median ICU stay was 2 days, and the median hospital stay was 10 days in both groups (Table 8).

**Table 8:** Postoperative outcomes of patients who underwent atrial septal defect closure or cardiac tumor resection

	ASD (n = 232)	Cardiac tumor (n = 126)
30-day mortality	0	1 (0.8%)
In-hospital mortality	0	2 (1.6%)
Reoperation for bleeding	2 (0.9%)	1 (0.8%)
Stroke	0	1 (0.8%)
Thoracotomy infection	0	0
Groin area infection	0	1 (0.8%)
Prolonged ventilation	1 (0.4%)	1 (0.8%)
Renal failure	1 (0.4%)	1 (0.8%)
New onset of AF	32 (13.8%)	14 (11.1%)
Heart block	1 (0.4%)	1 (0.8%)
Perioperative MI	0	0
Aortic dissection	1 (0.4%)	0
Acute limb ischemia	0	0
ICU stay (median, days)	2	2
Hospital stay (median, days)	10	10

ASD, atrial septal defect; AF, atrial fibrillation; MI, myocardial infarction; ICU, intensive care unit; Underlined values denote medians

### Three-year trends in MICS procedures (2021–2023)

**Fig. 2:**
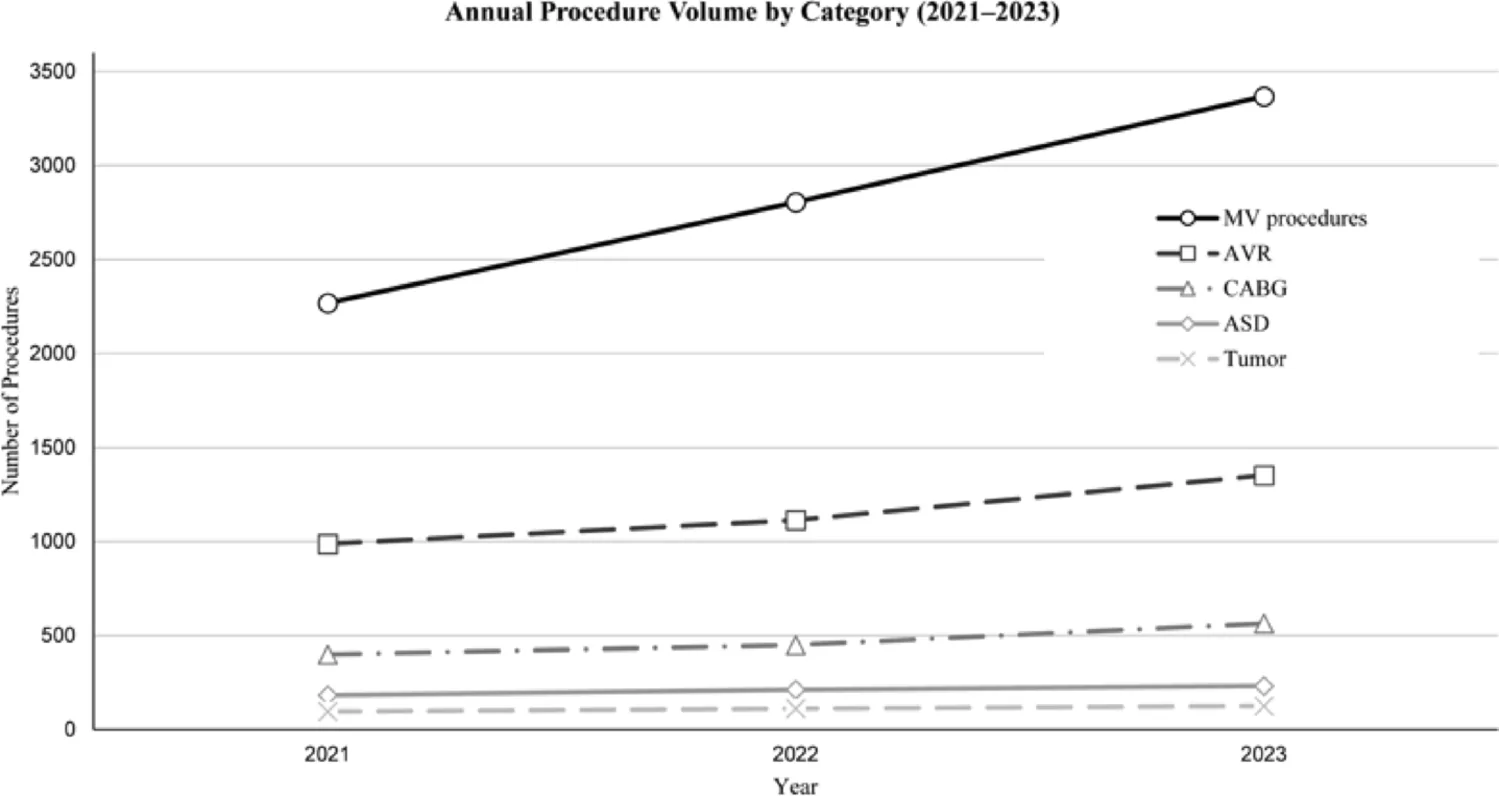
Overall annual procedure volume by category (2021–2023). Three-year trends in case volume for minimally invasive cardiac surgery (MICS) are shown across five major categories: mitral valve (MV) procedures, aortic valve replacement (AVR), coronary artery bypass grafting (CABG), atrial septal defect (ASD) closure, and cardiac tumor resection

**Fig. 3:**
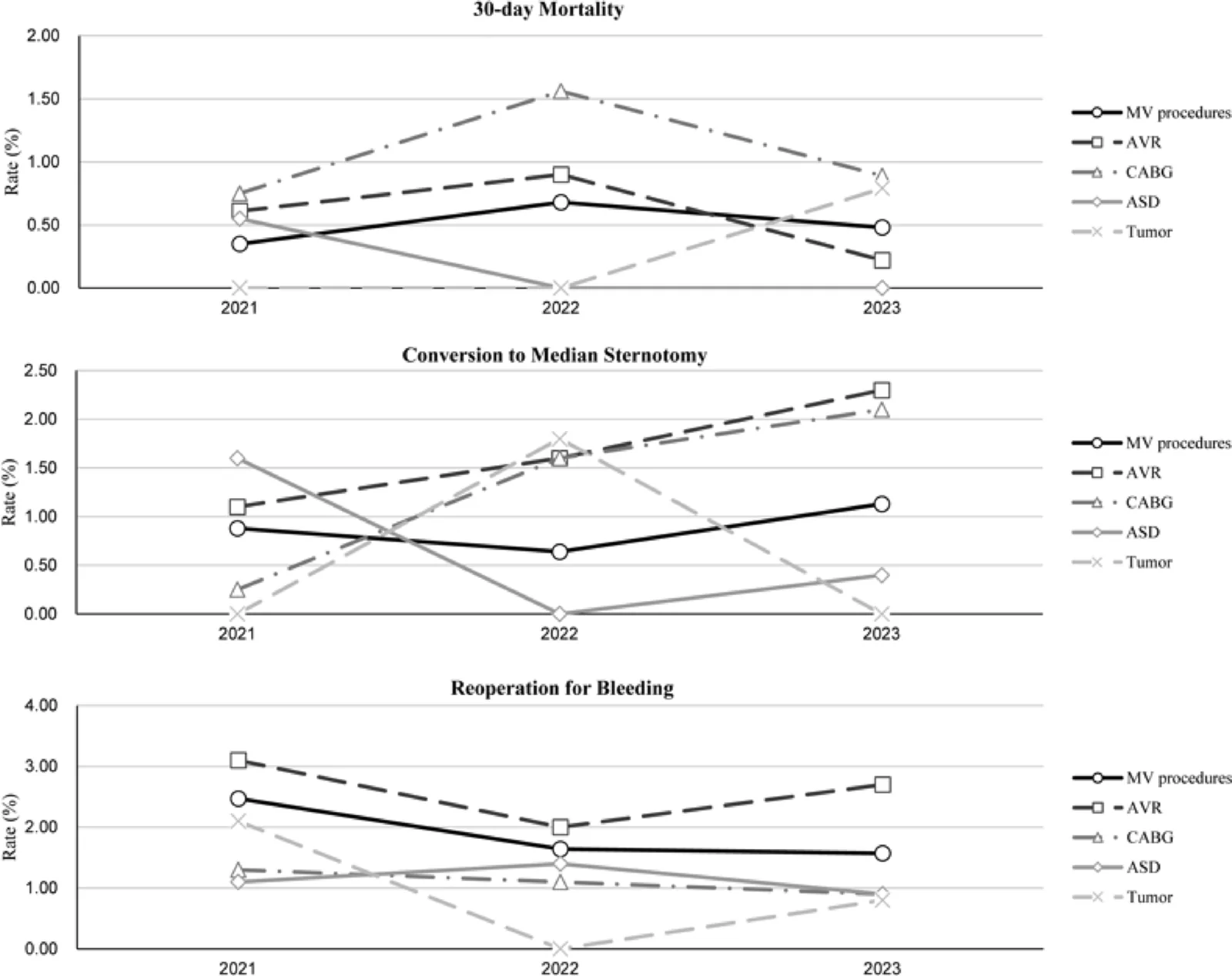
Overall early outcomes across major minimally invasive cardiac surgery (MICS) procedure categories (2021–2023). Three-year trends in early perioperative outcomes are shown across the five major procedure groups: mitral valve (MV) procedures, aortic valve replacement (AVR), coronary artery bypass (CABG), atrial septal defect (ASD) closure, and cardiac tumor resection. The upper panel shows the 30-day mortality, the middle panel shows conversion to median sternotomy, and the lower panel shows reoperation for bleeding

From 2021 to 2023, the annual volume of MICS increased in all five major procedure categories. MV surgery remained the most common procedure throughout the study period and showed the most marked increase; AVR and CABG also demonstrated steady upward trends. Although the number of ASD closures and cardiac tumor resections remained small, both categories increased gradually over time. Despite this overall increase in case volume, the early outcomes remained stable. The 30-day mortality and conversion to median sternotomy were low across all major categories, although slight increases in conversion were observed in the AVR and CABG groups in 2023. Reoperation for bleeding remained relatively infrequent throughout the study period (Figs. 2 and 3).

**Fig. 4:**
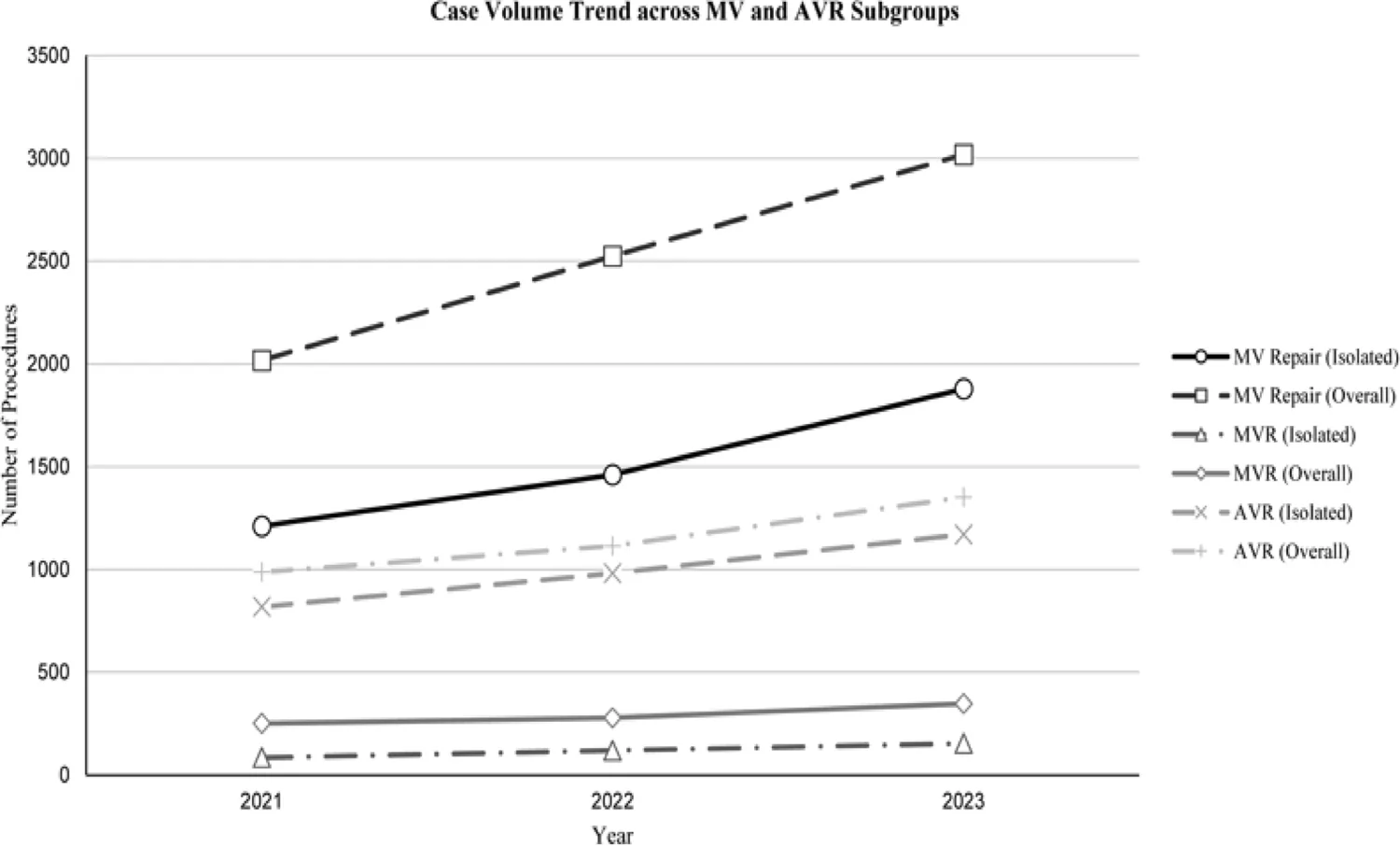
Case volume trends across mitral valve (MV) and aortic valve replacement (AVR) subgroups (2021–2023). Annual procedure volume stratified by isolated and overall cases for MV repair, mitral valve replacement (MVR), and AVR

**Fig. 5:**
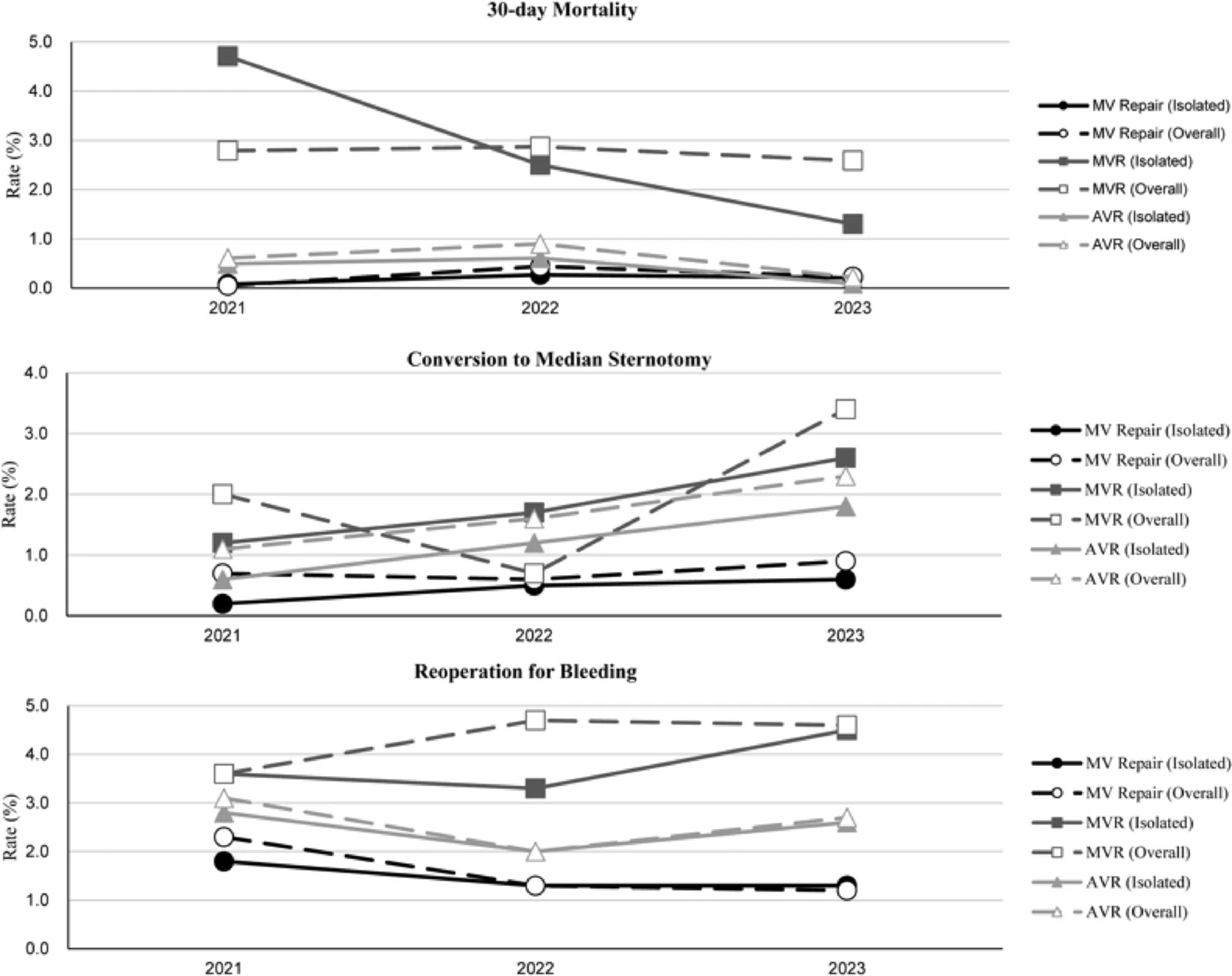
Early outcomes across mitral valve (MV) and aortic valve replacement (AVR) subgroups (2021–2023). Three-year trends in early perioperative outcomes are shown for isolated and overall MV repair, MVR, and AVR. The upper panel shows the 30-day mortality, the middle panel shows conversion to median sternotomy, and the lower panel shows reoperation for bleeding

Within the valve subgroups, the increase in MV surgery was primarily driven by MV repair, with both isolated and overall MV repair cases progressively increasing over the 3-year period. The number of isolated and overall AVR cases also increased steadily. In contrast, MVR accounted for the smallest proportion among the valve subgroups, although a gradual increase was observed in its proportion. In terms of early outcomes, isolated and overall MV repair, as well as AVR, consistently showed low 30-day mortality throughout the study period. In contrast, MVR exhibited higher 30-day mortality, conversion rates, and reoperation for bleeding than MV repair and AVR (Figs. 4 and 5).

**Fig. 6:**
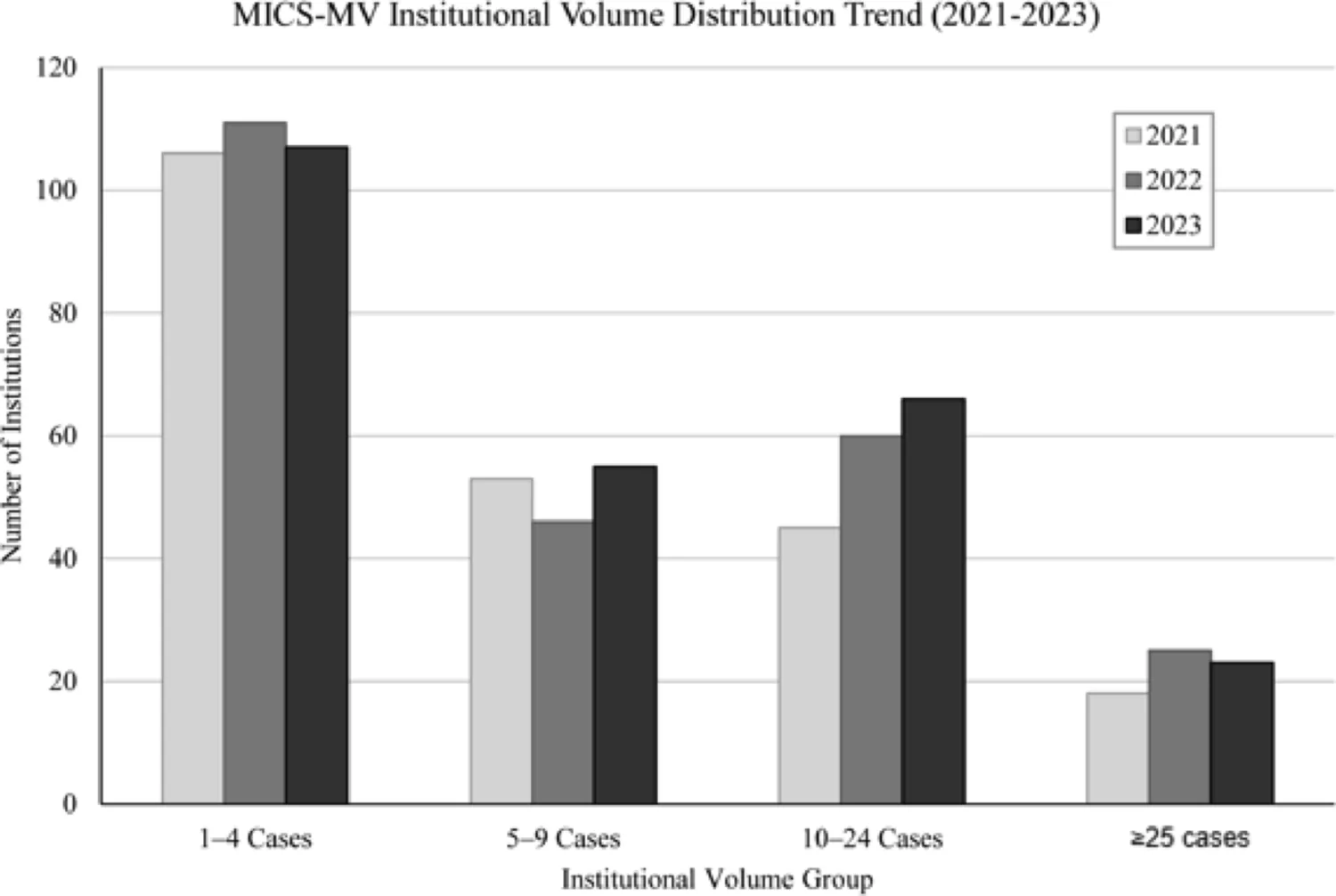
Institutional volume distribution of minimally invasive cardiac surgery mitral valve (MICS-MV) procedures (2021–2023). The number of institutions performing MICS-MV procedures is stratified into four categories according to annual case volume: 1–4, 5–9, 10–24, and ≥25 cases per year

**Fig. 7:**
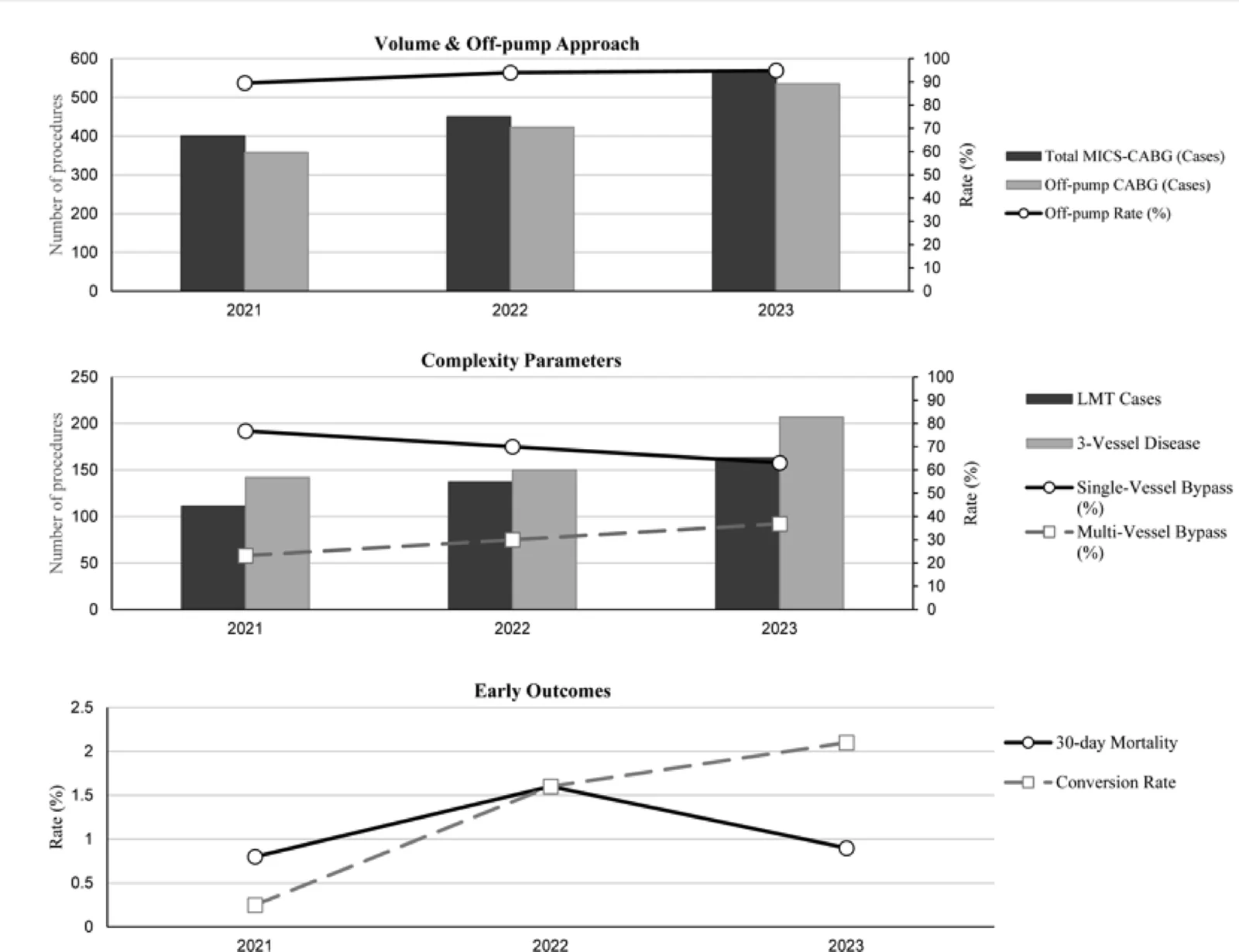
Trends in minimally invasive coronary artery bypass grafting (MICS-CABG) volume, complexity parameters, and early outcomes (2021–2023). Three-year trends in MICS-CABG volume, procedural complexity, and early outcomes are shown. The upper panel shows the total MICS-CABG volume, off-pump CABG volume, and the off-pump rate. The middle panel shows complexity parameters, including the number of cases with left main trunk (LMT) lesions and 3-vessel disease, as well as the proportions of single-vessel and multi-vessel bypass. The lower panel shows the 30-day mortality and conversion to median sternotomy

The institutional volume distribution of MV procedures also changed over time. Although low-volume centers performing 1 to 4 cases annually continued to account for a substantial proportion of institutions, the number of centers performing ≥10 cases annually increased over the study period, and the 5 to 9 case category also showed an overall increase in 2023 (Fig. 6). In MICS-CABG, the off-pump approach remained predominant, approaching 95% by 2023. Simultaneously, procedural complexity appeared to increase, as reflected by the decreasing proportion of single-vessel bypasses and increasing numbers of multi-vessel procedures, LMT lesions, and 3-vessel disease. Despite this trend, the mean JapanSCORE for MICS-CABG remained relatively stable at 2.5 ± 5.6 in 2021, 2.8 ± 4.3 in 2022, and 2.6 ± 3.7 in 2023, and the 30-day mortality remained below 2.0%, with conversion at approximately 2.0% throughout the study period (Fig. 7).

## Discussion

This nationwide report compiles the perioperative outcomes of minithoracotomy-based and related MICS procedures performed in Japan in 2023. Building on the findings of the 2021 and 2022 annual reports, the present analysis provides updated and comprehensive data across all major MICS categories, including MV surgery, AVR, CABG, ASD closure, and cardiac tumor resection [5, 6]. Importantly, when the present findings are viewed alongside the two previous annual reports, a consistent 3-year increase in case volume is evident across all major categories. In the broader national context, the cardiovascular surgical volume in Japan increased to 63,427 cases in 2023, and the number of procedures for valvular heart disease increased to 17,805 cases [7]. Within this changing landscape, updated nationwide MICS benchmarks remain highly relevant for quality assessment in contemporary clinical practice.

MV surgery remained the largest MICS category throughout the 3-year period and demonstrated the most marked growth. This increase was driven predominantly by MV repair. For isolated MV repair, the 30-day and in-hospital mortality rates in 2023 were exceptionally low, both at 0.2%. These results are consistent with recent randomized evidence suggesting that minithoracotomy for MV repair achieves safety comparable to that of conventional sternotomy [3]. In addition, isolated MV repair was associated with a median ICU stay of 2 days and a median hospital stay of 10 days in 2023, suggesting favorable early postoperative recovery in this subgroup. In comparison, the 2023 JATS annual report showed that the 30-day and in-hospital mortality rates for isolated MV repair in Japan were 0.7% and 1.1%, respectively [7]. For isolated MVR, MICS procedures resulted in 30-day and in-hospital mortality rates of 1.3% and 1.9%, respectively. Although MVR consistently showed higher 30-day mortality, conversion, and bleeding rates compared with MV repair across the study period, the 30-day and in-hospital mortality rates were substantially lower than the 3.7% and 6.9% reported for isolated MVR in the 2023 JATS report [7]. Notably, the conversion rate in the overall MVR cohort was 3.4% in 2023, which was higher than the corresponding rates in 2021 (2.0%) and 2022 (0.7%). Given the relatively small number of MVR cases, this increase should be interpreted cautiously; however, together with the persistently higher mortality and bleeding rates, it further indicates that MVR remained a higher-risk subgroup than MV repair in the present series. Another important finding is the shift in institutional distribution; although low-volume centers continued to account for a substantial proportion of institutions, the number of centers performing ≥10 cases per year increased over time [11]. Taken together, these data suggest that the MICS approach for MV disease in Japan has been implemented with favorable early outcomes during nationwide expansion and a shift in institutional case distribution.

For aortic valve surgery, the MICS case volume also increased steadily over time, reaching 1352 cases in 2023. Early outcomes remained highly favorable, particularly for isolated AVR, which had 30-day and in-hospital mortality rates of 0.1% and 0.3%, respectively. These values compare favorably with the rates of 1.6% and 2.6% for isolated AVR reported in the 2023 JATS annual report [7]. Notably, across the 3-year period, the rate of conversion to median sternotomy for isolated AVR increased from 0.6% in 2021 to 1.2% in 2022 and 1.8% in 2023. Although the overall mortality remained low throughout the study period, this upward trend suggests that technical challenges remain in contemporary MICS-AVR practice. This finding may reflect broader dissemination of the procedure, expansion to patients with more technically demanding anatomies, or both. Therefore, continued nationwide monitoring will be important for future annual evaluations. These consistent outcomes suggest that MICS-AVR is becoming an established sternum-sparing surgical option for selected patients.

The number of isolated MICS-CABG procedures also increased over the 3-year period, from 400 in 2021 to 564 in 2023. Simultaneously, the procedural profile became more complex. The off-pump approach remained predominant and approached 95% by 2023, whereas the proportion of single-vessel bypass decreased, and the number of procedures for LMT lesions and 3-vessel disease increased. Despite these changes in procedural complexity, predicted operative risk remained stable, and the 30-day and in-hospital mortality rates for MICS-CABG were 0.9% and 1.2% in 2023, respectively. For reference, in the 2023 JATS annual report, the 30-day and in-hospital mortality rates for primary elective off-pump CABG were 0.8% and 1.4%, respectively [7]. Thus, unlike in 2022, the early mortality outcomes of MICS-CABG in 2023 were broadly comparable to contemporary national off-pump CABG benchmarks [6]. Throughout the study period, MICS-CABG mortality remained below 2.0%, and the conversion rate to median sternotomy was 2.1% in 2023. These findings suggest that the expansion of MICS-CABG in Japan has not been limited to the simplest coronary anatomy and that acceptable early outcomes have been maintained even as procedural complexity has increased [12, 13].

For ASD closure and cardiac tumor resection via right minithoracotomy, the case volume also increased gradually over the 3-year period. Early outcomes in ASD remained excellent, with no 30-day or in-hospital deaths in 2022 or 2023. For cardiac tumor cases, the 30-day and in-hospital mortality rates in 2023 were 0.8% and 1.6%, respectively, compared with 0% and 1.0% in 2021, and 0% and 0% in 2022. Although this represents a numerical increase in mortality in 2023, the cohort remained relatively small, and the absolute number of events was limited in this study. Accordingly, this finding should be interpreted cautiously, and continued nationwide observation will be important in future annual evaluations. In the 2023 JATS annual report, the total number of cardiac tumor procedures nationwide was 660, including 587 benign and 73 malignant tumors [7], whereas the present MICS cohort included 126 procedures. These findings suggest that right minithoracotomy remains a feasible approach in selected ASD and cardiac tumor cases, although careful interpretation is necessary given the limited sample size.

The 3-year trends shown in this report are informative from the standpoint of program development. Across the major MICS categories, the procedure volume increased without an obvious deterioration in early outcomes at the national level. Thus, the expansion of MICS in Japan appears to have been accompanied not only by an increase in case numbers but also by a diversification of indications and procedural profiles. These temporal changes further support the value of continuous annual nationwide evaluations and benchmarking [9, 14]. Furthermore, the use of robot-assisted MICS has expanded alongside conventional approaches. In the JCVSD, the number of robot-assisted MV repairs increased from 532 in 2021 to 624 in 2022 and 816 in 2023. Although robot-assisted procedures were registered in the JCVSD, they were not included as a separate analytic group in the present JMICS report and therefore were not directly compared with non-robotic MICS. Nevertheless, a recent nationwide analysis from Japan showed perioperative outcomes comparable to those of the small right thoracotomy approach, with no meaningful difference in early mortality [15]. These findings suggest that robotic MICS is increasingly being incorporated into contemporary MICS practice in Japan, and more granular evaluation will be possible as additional MICS-specific parameters are collected from 2024 onward.

This study had several limitations. First, this annual report is based on registry data and focuses on early perioperative outcomes; as such, it does not address longer-term durability or late functional results. Second, although the temporal comparison from 2021 to 2023 is informative, the present analysis remains descriptive and does not account for potential differences in case selection, institutional practice, or unmeasured confounders. In particular, the relationship between institutional case volume and clinical outcomes represents an important next step that warrants a dedicated risk-adjusted analysis in future studies. Third, comparisons with the JATS annual report should be interpreted cautiously because the national report reflects broader surgical populations with varying risk profiles rather than an approach-specific MICS cohort. Finally, detailed MICS-specific information is limited within the current reporting framework. To address this issue, the JMICS and JCVSD collaborated to introduce 20 additional perioperative parameters specific to MICS, beginning in 2024. More recently, this set was streamlined to 17 items beginning in 2026 to improve data entry efficiency while retaining clinically useful information. These refinements are expected to enable more granular analyses in future nationwide studies.

In conclusion, 2023 nationwide data demonstrate the continued expansion of MICS activity in Japan across MV surgery, AVR, CABG, ASD closure, and cardiac tumor resection while maintaining generally favorable early outcomes. When viewed together with the 2021 and 2022 annual reports, the present findings indicate that the MICS program has expanded progressively over the past 3 years in terms of both volume and procedural scope. These updated nationwide results provide an important benchmark for ongoing quality improvement and continued evaluation of the role of MICS in contemporary cardiac surgery.

## Data Availability

The data used in this study were obtained from the Japan Cardiovascular Surgery Database (JCVSD) within the National Clinical Database (NCD) and are not publicly available due to data privacy restrictions.

## Abbreviations

ASD: Atrial septal defect
AVR: Aortic valve replacement
CABG: Coronary artery bypass grafting
ICU: Intensive care unit
JapanSCORE: Japan risk score for operative mortality
JATS: Japanese association for thoracic surgery
JCVSD: Japan cardiovascular surgery database
JMICS: Japanese association for minimally invasive cardiac surgery
LMT: Left main trunk
MICS: Minimally invasive cardiac surgery
MV: Mitral valve
MVR: Mitral valve replacement
STS: Society of thoracic surgeons

## References

[1] Nishi H, Miyata H, Motomura N, Toda K, Miyagawa S, Sawa Y, et al. Propensity-matched analysis of minimally invasive mitral valve repair using a nationwide surgical database. Surg Today. 2015;45(9):1144–52. 10.1007/s00595-015-1210-7. 26112649

[2] Nissen AP, Miller CC 3rd, Thourani VH, Woo YJ, Gammie JS, Ailawadi G, et al. Less invasive mitral surgery versus conventional sternotomy stratified by mitral pathology. Ann Thorac Surg. 2021;111(3):819–27. 10.1016/j.athoracsur.2020.05.145. 32717233

[3] Akowuah EF, Maier RH, Hancock HC, Kharatikoopaei E, Vale L, Fernandez-Garcia C, et al. Minithoracotomy vs conventional sternotomy for mitral valve repair. JAMA. 2023;329(22):1957–66. 10.1001/jama.2023.7800. PMC10265311 37314276

[4] Holzhey DM, Seeburger J, Misfeld M, Borger MA, Mohr FW. Learning minimally invasive mitral valve surgery. Circulation. 2013;128(5):483–91. 10.1161/CIRCULATIONAHA.112.001402. 23804253

[5] Shimokawa T, Kumamaru H, Motomura N, Nishi H, Nakajima H, Kamiya H, et al. Minimally invasive cardiac surgeries in 2021: annual report by Japanese society of minimally invasive cardiac surgery. Gen Thorac Cardiovasc Surg. 2025;73(2):88–95. 10.1007/s11748-024-02066-2. 39085564

[6] Shimokawa T, Kumamaru H, Motomura N, Nishi H, Nakajima H, Kamiya H, et al. Minimally invasive cardiac surgeries in 2022: annual report by Japanese society of minimally invasive cardiac surgery. Gen Thorac Cardiovasc Surg. 2026;75(5):510–7. 10.1007/s11748-025-02225-z. PMC13139202 41417160

[7] Committee for Scientific Affairs, The Japanese Association for Thoracic Surgery, Yoshimura N, Sato Y, Takeuchi H, Abe T, et al. Thoracic and cardiovascular surgeries in Japan during 2023: annual report by the Japanese Association for Thoracic Surgery. Gen Thorac Cardiovasc Surg. 2025;73(7):526–65. 10.1007/s11748-025-02128-z. PMC12174170 40377897

[8] Takamoto S, Motomura N, Miyata H, Tsukihara H. Current status of cardiovascular surgery in Japan, 2013 and 2014: a report based on the Japan cardiovascular surgery database (JCVSD). 1: Mission and history of JCVSD. Gen Thorac Cardiovasc Surg. 2018;66(1):1–3. 10.1007/s11748-017-0843-4. 29134534

[9] Miyata H, Motomura N, Murakami A, Takamoto S, Japan Cardiovascular Surgery Database. Effect of benchmarking projects on outcomes of coronary artery bypass graft surgery. J Thorac Cardiovasc Surg. 2012;143(6):1364–9. 10.1016/j.jtcvs.2011.07.010. 22595627

[10] Miyata H, Tomotaki A, Motomura N, Takamoto S. Operative mortality and complication risk model for all major cardiovascular operations in Japan. Ann Thorac Surg. 2015;99(1):130–9. 10.1016/j.athoracsur.2014.07.038. 25442990

[11] Heuts S, Hjij W, Kawczynski MJ, Olsthoorn JR, Tjon Joek Tjien A, van Kuijk S, et al. Defining centres of expertise for minimally invasive mitral valve surgery. Heart. 2025;111(20):984–91. 10.1136/heartjnl-2024-325048. 40348408

[12] Sharaf M, Zittermann A, Sunavsky J, Gilis-Januszewski T, Rojas SV, Götte J, et al. Early and late outcomes after minimally invasive direct coronary artery bypass vs full sternotomy off-pump coronary artery bypass grafting. Front Cardiovasc Med. 2024;11:1298466. 10.3389/fcvm.2024.1298466. PMC10914960 38450373

[13] Rufa MI, Ursulescu A, Ahad S, Nagib R, Albert M, Ghinescu M, et al. Medium- and long-term outcomes of 597 patients following minimally invasive multi-vessel coronary off-pump bypass surgery. J Clin Med. 2025;14(5):1707. 10.3390/jcm14051707. PMC11900377 40095802

[14] Beckmann A, Meyer R, Eberhardt J, Gummert J, Falk V. German heart surgery report 2024. Thorac Cardiovasc Surg. 2025;73(8):595–608. 10.1055/a-2737-6756. 41330389

[15] Watanabe G, Kumamaru H, Kinukawa N, Shibata T, Shiose A, Takagi Y, et al. Successful launch of robot-assisted mitral valve repair in Japan under universal health coverage. J Thorac Cardiovasc Surg. 2025;169(4):1242–50. 10.1016/j.jtcvs.2024.05.014. 38802045

